# Is metabotropic glutamate receptor 5 upregulated in prefrontal cortex in fragile X syndrome?

**DOI:** 10.1186/2040-2392-4-15

**Published:** 2013-05-24

**Authors:** Talakad G Lohith, Emily K Osterweil, Masahiro Fujita, Kimberly J Jenko, Mark F Bear, Robert B Innis

**Affiliations:** 1 Molecular Imaging Branch, National Institute of Mental Health, 10 Center Drive, Bethesda, MD 20892-1026, USA; 2 Department of Brain and Cognitive Sciences, Howard Hughes Medical Institute, The Picower Institute for Learning and Memory, Massachusetts Institute of Technology, Cambridge, MA, 02139, USA

**Keywords:** Fragile X mental retardation protein, Fragile X syndrome, Glutamate receptor, mGluR5, Receptor density, Receptor expression

## Abstract

**Background:**

Fragile X syndrome (FXS) is a common inherited form of intellectual disability caused by loss of function of the fragile X mental retardation protein. Recent animal studies suggest that upregulated downstream signaling by metabotropic glutamate receptor 5 (mGluR5) might be an important mechanism for cognitive and behavioral abnormalities associated with FXS. However, mGluR5 density in human FXS remains unknown.

**Methods:**

Receptor binding and protein expression were measured in the postmortem prefrontal cortex of 14 FXS patients or carriers and 17 age- and sex-matched control subjects without neurological disorders. *In-vitro* binding assays were performed using [^3^H]-labeled 3-methoxy-5-pyridin-2-ylethynylpyridine (MPEPy), a selective and high-affinity negative allosteric modulator of mGluR5, to measure receptor density and the radioligand’s dissociation constant, which is inversely proportional to affinity. Immunoblotting was also performed, to measure mGluR5 protein expression.

**Results:**

The mGluR5 density increased with marginal significance (+16%; *P* = 0.058) in the prefrontal cortex of FXS patients or carriers compared with matched healthy controls. No significant change in dissociation constant (-4%; *P* = 0.293) was observed. Immunoblotting found a significant elevation (+32%; *P* = 0.048) in mGluR5 protein expression.

**Conclusions:**

Both mGluR5 binding density and protein expression were increased in the brains of FXS patients or carriers, but only expression was significantly different, which could be because of the small sample size and moderate variability. Another important caveat is that the effects of psychotropic medications on mGluR5 expression are largely unknown. Future *in-vivo* measurement of mGluR5 with positron emission tomography might characterize the role of this receptor in the pathophysiology of FXS and facilitate trials of mGluR5-oriented treatments for this disorder.

## Background

Fragile X syndrome (FXS), the most common cause of inherited intellectual disability, is associated with multiple cognitive, behavioral, and neuropsychiatric abnormalities [1]. The disorder is caused by expansion of the CGG trinucleotide repeat in the 5′ untranslated region of the fragile X mental retardation 1 (*FMR1*) gene located at the distal end of the long arm of the X chromosome. The length of repeat expansion determines the expression of FXS disorder: alleles containing ≤44 repeats are considered ‘normal’, those containing between 44 and 54 repeats form a ‘gray zone’, those containing between 55 and 200 repeats are ‘premutation carriers’, and alleles with ≥200 repeats are ‘full mutations’ [2]. Only the full mutation leads to hypermethylation of the *FMR1* promoter and consequent transcriptional silencing of the *FMR1* gene that encodes fragile X mental retardation protein (FMRP) [3]. FMRP is a cytoplasmic RNA-binding protein known to repress the translation of specific messenger RNAs at neuronal synapses, thereby regulating expression of various synaptic proteins [4,5]. FMRP thus plays a major role in synaptic signal transduction, and its loss dysregulates neuronal signaling pathways. One of the key signaling pathways believed to be dysregulated in FXS as the result of a loss of FMRP is mediated by metabotropic glutamate receptors (mGluRs), particularly subtype 5 (mGluR5). Studies have demonstrated that dysregulated mGluR5 signaling results in uncontrolled activation of synaptic protein synthesis, owing to the loss of normal repressor action of FMRP on protein synthesis [6,7].

Recent studies using various animal models of FXS have suggested that many features of the FXS phenotype, including behavioral abnormalities, cognitive deficits, and altered dendritic spines, may be attributable to exaggerated mGluR5 signaling. Some studies that sought to suppress mGluR5 signaling by genetic knockdown or by using negative allosteric modulators of mGluR5, such as 2-methyl-6-(phenylethynyl)-pyridine (MPEP), corrected most of these phenotypic features in mouse models of FXS [8,9]. Although evidence from rodent studies suggests that mGluR5 signaling is increased in FXS, mGluR5 density has yet to be examined in the postmortem brain tissue of individuals with FXS. Answering this question is important because measuring mGluR5 levels could allow categorization of FXS patient subgroups based on receptor density and, relatedly, help identify those FXS patients who might be promising candidates for treatment with mGluR5 allosteric modulators.

To determine whether mGluR5 levels are altered in FXS, we measured mGluR5 receptor density via radioligand binding assays, and expression levels via Western blotting. These measures were ascertained in the postmortem brain tissue of FXS patients and premutation carriers, and compared with measures ascertained in age- and sex-matched healthy controls.

## Methods

### Human postmortem tissues

Postmortem human brain tissue from the prefrontal or frontal cortex was provided by the National Institute of Child Health & Human Development Brain and Tissue Bank for Developmental Disorders, University of Maryland, Baltimore, MD, USA, and by the Hagerman Laboratory, Department of Biochemistry and Molecular Medicine, University of California, Davis, CA, USA. As per US regulation 45 CFR 46, these postmortem specimens from deceased subjects were exempted by the Office of Human Subjects for ethical approval and did not need subject consent for use in the study. Tissue samples were available for 14 white male FXS patients or carriers, of which one had fragile X-associated tremor ataxia syndrome (FXTAS) and 17 white male healthy controls; the demographic characteristics of the samples are presented in Table 1. The FXS patients or carrier samples and the control samples were matched for age and postmortem interval; other demographic differences were considered insignificant for this study. Medication status before death was not available for many of the samples. Because only four samples from FXS carriers were available, those four samples were pooled with FXS patient samples for analysis purposes.

**Table 1:** Demographic data for human brain tissue samples

**Case**^**a**^	**Diagnosis**	**CGG repeats**	**Age (years)**	**Postmortem interval (hours)**	**Cause of death**	**Central nervous system drug use**^**b**^
UMB-1421	Fragile X	563^d^, 297^e^	69	17	Cancer	NA
UMB-1938	Fragile X	NA	62	26	Hypertensive cardiovascular disease	NA
UMB-4751	Fragile X carrier	88	21	5	Pulmonary edema or pneumonia	Antiepileptics
UMB-4806	Fragile X	NA	9	22	Cardiac arrest	Clonidine
UMB-5006	Fragile X carrier	150	85	5	Complication of disorder	NA
UMB-5319	Fragile X	NA	71	17	Complication of disorder	NA
UMB-4664	Fragile X carrier	100	71	3	Complication of disorder	NA
UMB-5212	Fragile X carrier (FXTAS)	NA	80	12	Complication of disorder	NA
1031-08-GP	Fragile X	436	57	20	Food choking	Antipsychotics
JS-03	Fragile X	NA	25	16	NA	NA
1005-06-CB/XX	Fragile X	NA	55	-	NA	NA
1018-10-RH	Fragile X	NA	60	55	NA	NA
1031-09-LZ	Fragile X	429^d^, 340 – 440^e^	64	12	Cancer	None
1033-08-WS	Fragile X	> 339^d^	78	18	Chronic obstructive pulmonary disease or cardiac block	Antiepileptics
UMB-1442	Healthy	NA	22	7	Multiple injuries	NA
UMB-5082	Healthy	NA	68	19	Arteriosclerotic cardiovascular disease	None
UMB-5123	Healthy	NA	61	24	Arteriosclerotic cardiovascular disease	NA
UMB-1103	Healthy	NA	21	7	Accident, multiple injuries	NA
UMB-1674	Healthy	NA	8	36	Hyperthermia and drowning	NA
UMB-5182	Healthy	NA	86	23	Hypertensive cardiovascular disease	NA
UMB-1104	Healthy	NA	35	12	Accident, multiple injuries	Alcohol
UMB-4593	Healthy	NA	33	8	Cardiac arrhythmia	NA
UMB-4598	Healthy	NA	45	6	Dilated cardiomyopathy	NA
UMB-4735	Healthy	NA	73	21	Chronic obstructive pulmonary disease	NA
UMB-5171	Healthy	NA	79	5	Chronic obstructive pulmonary disease or peripheral vascular disease	None
UMB-1039	Healthy	NA	49	9	Accident, multiple injuries	NA
UMB-1227	Healthy	NA	52	16	Pulmonary embolism	NA
UMB-1228	Healthy	NA	47	13	Arteriosclerotic cardiovascular disease	NA
UMB-1570	Healthy	NA	48	14	Arteriosclerotic cardiovascular disease	NA
UMB-1907	Healthy	NA	54	17	Accident, multiple injuries	NA
UMB-4915	Healthy	NA	49	5	Arteriosclerotic cardiovascular disease	NA
**Number of subjects ( *n *)**		**Diagnosis**	**Age (years)**^**c**^		**Postmortem interval (hours)**^**c**^	
			**Mean**	**Range**	**Mean**	**Range**
14		Fragile X (*n *= 10) or carrier (*n *= 4)	58	9 to 85	17	3 to 55
17		Healthy	49	8 to 86	14	5 to 36

FXTAS, fragile X-associated tremor ataxia syndrome; NA, not available; ^a^all brain tissues were from white male subjects and from prefrontal cortex except for case 1033-08-WS, which was from frontal cortex; ^b^at time of death; ^c^no significant difference was found in age (*t *= 1.11, d*f *= 29, *P *= 0.276) or postmortem interval (*t *= 0.83, d*f *= 28, *P *= 0.411) between FXS patients or carriers and healthy controls; ^d^hypermethylated repeats; ^e^unmethylated repeats.

### Tissue preparation

Tissue samples were homogenized using a mortar and pestle in fresh ice-cold, 50 mM Tris–HCl buffer (1:10 w/v) completing 3 × 10 passes with cooling on ice between homogenizations. Homogenates were centrifuged at 20,000 *g* and 4°C for 25 minutes, followed by removal of the supernatant. Pellets were then resuspended in fresh ice-cold 50 mM Tris–HCl buffer and centrifuged again at the same settings. Pellets were resuspended in fresh ice-cold 50 mM Tris–HCl buffer at a protein concentration of approximately 1 mg of protein/mL. Aliquots were stored in a freezer at -80°C until further use. Protein concentrations were determined using the Bradford protein assay (Bio-Rad, Hercules, CA), and absorption was read at 595 nm.

### Competition binding assays

An ethanolic solution of [^3^H]-labeled 3-methoxy-5-pyridin-2-ylethynylpyridine (MPEPy), a selective and high-affinity negative allosteric modulator of mGluR5, was purchased from American Radiolabeled Chemicals Inc. (St. Louis, MO) at a specific activity of 2.96 TBq/mmol and a radiochemical purity of 99%. Nonradioactive MPEPy was purchased from Tocris Bioscience (Ellisville, MO). The chemical purity of nonradioactive MPEPy was 100%, as determined by high performance liquid chromatography.

The specific binding, mGluR5 receptor density (*B*_max_), and the radioligand’s dissociation constant (*K*_D_), which is inversely proportional to affinity, were determined in human brain tissue homogenates using standard, steady-state homologous competition binding assays [10,11] using 0.48 nM [^3^H]MPEPy and concentrations of unlabeled MPEPy ranging from 0.1 nM to 1 μM. Nonspecific binding was determined in the presence of 10 μM of unlabeled ligand. Assays were performed for each tissue sample in triplicate with 0.1 mg protein per assay tube in a final volume of 300 μL, and incubated with shaking for 2 hours at room temperature. After incubation, assay reaction was terminated by filtration using a cell harvester (Model# M-48T, Brandel Inc., Gaithersburg, MD) through Whatman GF/B filters (Brandel Inc., Gaithersburg, MD) followed by 3 × 1 mL washes with ice-cold 50 mM Tris–HCl buffer. Whatman GF/B filters were preincubated with 0.5% polyethylenimine for 30 minutes before filtration. Scintillation fluid (Ultima Gold, PerkinElmer, Downers Grove, IL) was added at 4 mL/vial; vials were counted on a Tri-Carb 3100TR liquid scintillation counter (PerkinElmer, Downers Grove, IL) for five minutes each.

### Western blotting

Cortical tissue samples were boiled in Laemmli sample buffer and 20 μg of total protein resolved on 10% SDS PAGE gels. All gels were loaded with an equal number of control and FXS samples, and these were loaded in an alternating fashion, to distribute both genotypes equally across the gel. Gels were transferred to nitrocellulose membranes (Bio-Rad, Hercules, CA) and stained for total protein using the Memcode staining kit (Pierce Biotechnology, Rockford, IL, USA). Immunoblotting was performed using 2 μg/mL primary antibody to mGluR5 (Neuromics, Northfield, MN, USA), HRP-conjugated secondary antibody (GE Healthcare, Milwaukee, WI, USA), and ECL plus (GE Healthcare, Milwaukee, WI, USA).

### Data and statistical analysis

To determine the receptor density and dissociation constant, specific binding from triplicate measurements of each sample of competition assay data was analyzed using the iterative nonlinear regression curve-fitting software in GraphPad Prism software (version 5.0, GraphPad Software Inc., San Diego, CA, USA). The equation for specific binding is:

$$\text{Specific}\;\text{binding}=\frac{B_{\text{max}}\cdot \left[\text{Hot}\right]}{\left[\text{Hot}\right]+\left[\text{Cold}\right]+K_{D}}$$

[Hot] and [Cold] represents [^3^H]MPEPy and unlabeled MPEPy respectively. One-site and two-site homologous competition models were fitted to the data using the least-squares algorithm, and the model was selected using the *F* test. The null hypothesis, that the data would fit a one-site model, was rejected if the *P* value was less than 0.05. The two-site model was rejected if the receptor density from low-affinity sites had inappropriately high values [12].

The level of mGluR5 expression was measured by densitometry using Quantity One software (Bio-Rad), and normalized to a one-dimensional line-scan of the total protein Memcode signal in the same lane. To correct for blot-to-blot variance, each signal was normalized to the average signal of all lanes on the same blot. Outliers were removed from data analysis if the final values fell outside plus or minus two standard deviations. All gels were loaded and analyzed blind to genotype and treatment.

Data were analyzed for normal distribution by the Shapiro-Wilk normality test using SPSS Statistics (Version 17.0, SPSS Inc., Chicago, IL, USA). If the samples were normally distributed, statistical comparisons were performed using the two-tailed unpaired Student’s *t*-test; otherwise, the nonparametric Mann–Whitney test was performed to obtain *P* values. Correlations between receptor density and expression levels were examined with Pearson’s correlation test. A *P* value of less than 0.05 was considered statistically significant, between 0.05 to 0.1 as marginally significant and more than 0.1 as nonsignificant.

## Results

### Density and expression levels of mGluR5 were marginally higher in prefrontal cortex of FXS patients or carriers

**Figure 1:**
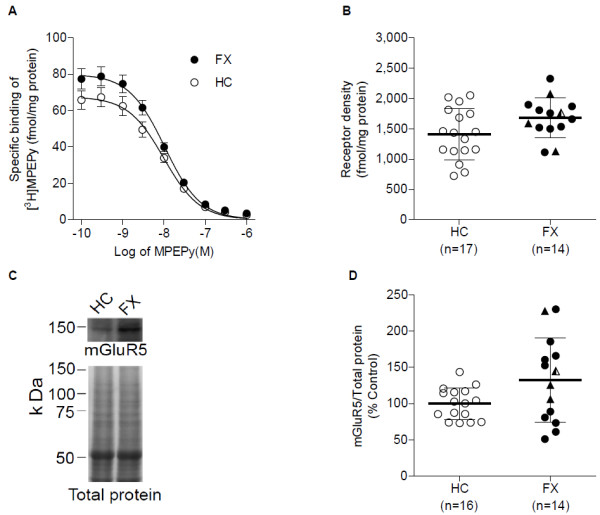
**mGluR5 receptor density and expression in prefrontal cortex of FXS individuals and healthy controls. **(**A**) Binding curves from homologous competition binding of 0.48 nM of [^3^H]MPEPy to membrane preparation from FXS and control subject samples at concentrations of unlabeled MPEPy ranging from 0.1 nM to 1 μM. Individual binding curves were obtained from the average of triplicate measurements for each unlabeled ligand concentration. Data represent mean ± standard error in the mean from 14 FXS patients or carriers (FX) and 17 healthy controls (HC). At low concentration of unlabeled ligand, specific binding was higher for FXS than control samples. (**B**) Results of unpaired *t*-test to compare the two groups. mGluR5 density tended to be higher (+16%; *P* = 0.058) in FXS patients than in the control group. Data represent mean ± standard deviation. Solid triangles (▲) in the FX group indicate the location of three FXS carriers; semisolid triangles () indicate the location of a carrier with FXTAS. (**C**) Representative immunoblot for mGluR5. The mGluR5 band intensity was stronger for the FXS than control subject. Total protein stain of the same lanes confirmed equal-protein loading. (**D**) Average mGluR5: total protein ratio normalized to control subjects. The ratio was high and marginally significant (+32%; *P *= 0.048) for the FXS group compared with controls. Data represent mean ± standard deviation. Solid triangles (▲) in the FX group indicate the location of three FXS carriers; semisolid triangles () indicate the location of a carrier with FXTAS.

The binding curves generated from homologous competition binding assays performed on postmortem prefrontal cortex samples of 14 FXS patients or carriers and 17 matched controls demonstrated that at low concentrations of unlabeled MPEPy, specifically bound [^3^H]MPEPy was higher in prefrontal cortex samples from the FXS group than the control group (Figure 1A). The binding curves best fit the one-site homologous model, which was used to determine binding measures. The mGluR5 receptor density increased with marginal significance (+16%; *t* = 1.97, d*f* = 29, *P* = 0.058) for FXS patients or carriers, as compared with control samples (Figure 1B, Table 2). The dissociation constant did not differ significantly between groups (-4%; Mann–Whitney U = 92.0, *P* = 0.293). In addition, Western blot analyses consistently revealed a stronger mGluR5 band corresponding to a molecular mass of 150 kDa in FXS patients or carriers (Figure 1C), with the average mGluR5: total protein ratio higher and significant (+32%; *t* = 2.07, d*f* = 28, *P* = 0.048) for FXS patients or carriers (mean = 1.32, standard deviation = 0.16) than for control samples (mean = 1.0, standard deviation = 0.05; Figure 1D). When the four FXS carriers were excluded from the analysis, both mGluR5 density and expression were higher (+17%; *t* = 1.88, d*f* = 25, *P* = 0.071 and +25%; *t* = 1.51, d*f* = 24, *P* = 0.147, respectively), but only mGluR5 density was marginally significant between FXS patients and control samples.

**Table 2:** Binding parameters from homologous competition binding assays

**Binding parameters**	**Healthy**	**Fragile X**		
	**(*n *= 17)**^**a**^	**(*n *= 14)**^**a**^	**% change**	***P***
Receptor density (fmol/mg protein)	1410 ± 421	1683 ± 372	16	0.058
Dissociation constant (nM)	10.6 ± 4.0	10.2 ± 1.6	-4	*0.293*^**b**^
Binding potential (receptor density/dissociation constant)	14 ± 5	17 ± 4	17	0.092

^a^Values represent mean ± standard deviation; ^b^*P *value determined from nonparametric Mann–Whitney test.

### Binding assay and immunoblot data correlated positively but were independent of age or postmortem interval

**Figure 2:**
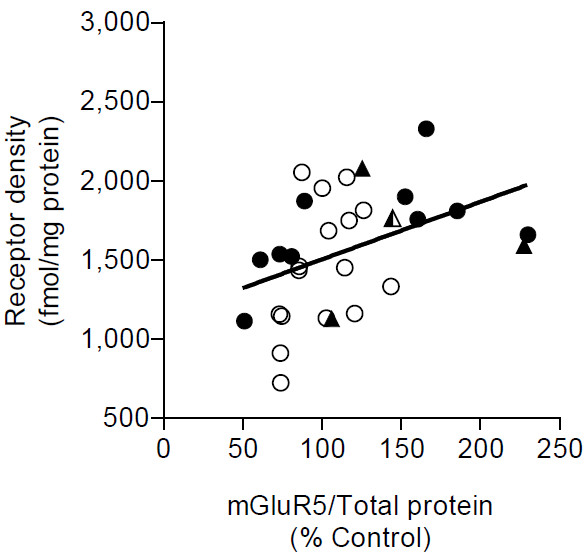
**Correlation between mGluR5 density and expression in prefrontal cortex of FXS individuals and healthy controls. **mGlur5 density by binding assay and expression by Western blotting positively correlated with a Pearson *r *= 0.43 and *P* = 0.018. Healthy controls and FXS full mutations are shown by open (○) and closed circles (●) respectively. The three FXS carriers and one carrier with FXTAS are shown by solid (▲) and semisolid () triangles, respectively. The regression line was determined using linear regression.

Pearson’s correlation analysis showed a significant positive correlation (*r* = 0.43; *P* = 0.018) between mGluR5 density values and expression levels from corresponding tissue samples (Figure 2). In addition, individually the correlation was positive and similar but not significant for both FXS patients or carriers (*r* = 0.42; *P* = 0.136) and control (*r* = 0.41; *P* = 0.114) subjects. However, receptor density and mGluR5 expression levels from both FXS patients or carriers and control subjects did not significantly correlate with age (mGluR5 density: *r* = -0.36, *P* = 0.203 and *r* = 0.13, *P* = 0.609; mGluR5 expression: *r* = -0.35, *P* = 0.216 and *r* = 0.17, *P* = 0.539 for FXS and controls, respectively) or postmortem interval (mGluR5 density: *r* = 0.26, *P* = 0.395 and *r* = -0.30, *P* = 0.249; mGluR5 expression: *r* = 0.23, *P* = 0.444 and *r* = 0.17, *P* = 0.525 for FXS and controls, respectively), indicating that mGluR5 receptor density and expression level occurred independently of age or postmortem interval.

## Discussion

Using *in-vitro* radioligand binding assays and Western blotting, we found a marginally significant increase in mGluR5 receptor density and a statistically significant increase in mGluR5 expression from the postmortem prefrontal cortex of FXS patients or carriers, compared with age- and sex-matched controls without neurological disorders. Highly significant differences in mGluR5 density could not be measured, mainly because of the limited availability of postmortem human brain tissues from FXS patients; nevertheless, the results raise the important question of whether mGluR5 density is indeed increased in the brains of individuals with FXS. While numerous preclinical studies have linked increased mGluR5 receptor signaling to various syndromic features of FXS [6,7], this study is the first to measure mGluR5 receptor density or expression levels directly in the human brain of individuals with FXS or carriers. It is important to note, however, that several reports have described changes in mGluR5 expression in patients with autism or in FXS model mice [13-15].

Notably, FXS is the most common single-gene cause of autism, accounting for 2 to 6% of all cases of autism, and numerous studies suggest that similar sets of proteins are dysregulated in both autism and FXS, including mGluR5 [14,15]. For instance, Fatemi and colleagues [13] recently reported a statistically significant increase in mGluR5 protein levels in postmortem tissue samples from the superior frontal cortex of autistic children, a finding that was not significant in autistic adults. Interestingly, the same study also found significantly lower levels of FMRP in autistic adults, despite the fact that no records indicated that any of the individuals were tested for or diagnosed with FXS. Considering that autism and FXS are intertwined at the molecular level [15], the findings of the autism study are in agreement with the significantly increased mGluR5 expression observed in the present study in human prefrontal cortex of individuals with FXS.

Reports of mGluR5 expression levels in brain samples from animal models of FXS are limited. Giuffrida and colleagues [16] recently found reduced mGluR5 expression in the detergent insoluble fraction of synaptic plasma membranes isolated from forebrains of *Fmr1* knockout mice compared with wild-type mice; however, the mRNA and total mGluR5 protein expression in total membrane protein preparation were comparable between the two mouse groups. The study attributed the altered mGluR5 distribution to a complex interaction with Homer scaffolding proteins. Another study found that mGluR5 levels were significantly reduced in the lateral geniculate nucleus of *Fmr1* KO mice [17]. In the *Fmr1* KO hippocampus, however, Western blot analysis revealed no difference in mGluR5 protein expression [8]. In contrast to these reports, our results indicated significantly elevated expression of mGluR5 in human prefrontal cortex samples of FXS patients or carriers compared with healthy controls. This discrepancy might be due to several key factors, including differences between species, between the brain regions studied, and between experimental procedures.

Presently, we can only speculate about the mechanisms that might explain the increased mGluR5 levels observed in prefrontal cortex of FXS patients or carriers. The absence of FMRP in FXS has been implicated for both decreases and increases of different mRNA pools, owing to translational repression or activation of diverse mRNAs [5]. Although FMRP is not believed to be directly associated with *Grm5* mRNA, its association with other mRNAs may indirectly upregulate mGluR5 levels [18]. However, overexpression of mGluR5 secondary to absence of FMRP may lead to reduced 2-amino-3-(5-methyl-3-oxo-1,2- oxazol-4-yl)propanoic acid/N-methyl-D-aspartate receptors as well as alterations in several other signaling molecules; thus, FXS is currently considered a disease of dysregulated neuronal signaling [5].

Preclinical studies in FXS model mice suggest that synaptic protein synthesis is dysregulated by hypersensitivity to mGluR5 signaling in FXS. Indeed, reducing mGluR5 signaling by genetic knockdown or by administering mGluR5 negative allosteric modulators, such as MPEP, improved cognitive and behavioral measures in a mouse model of FXS [8,9]. Negative allosteric modulators of mGluR5 are presently being considered as potential treatments for FXS. In a pilot, open-label, single-dose, Phase I study with fenobam, a negative allosteric modulator of mGluR5 comparable to MPEP, 9 of 12 subjects exhibited calmed behavior within one hour of dosing, and 6 of 12 subjects had improved prepulse inhibition [19]. Another trial administered AFQ056, an mGluR5 negative allosteric modulator, for several weeks. Although the overall therapeutic efficacy in FXS was negative, a *post-hoc* analysis found significant therapeutic efficacy in 7 patients who had full *FMR1* promoter methylation, but no response in 18 patients with partial promoter methylation [5,20]. Full methylation would be expected to cause complete silencing of the *FMR1* gene with minimal expression of FMRP (comparable to the mouse KO model), whereas partial methylation would be associated with variable FMRP levels; this could perhaps affect the degree of mGluR5 hyperactivation and, thus, treatment response. Future treatment trials are likely to prospectively separate patients with and without hypermethylation of the *FMR1* gene.

This study was associated with several limitations. First, the increased mGluR5 binding and expression observed in human prefrontal cortex samples from individuals with FXS patients or carriers might not reflect actual disease status; instead, it could result from treatment with antipsychotic medications. FXS patients are commonly treated with antipsychotic drugs in childhood and early adulthood, though usually not in late adulthood [21]. Medication status, which might have helped elucidate this issue, was not available for most of these tissue samples. Nevertheless, one study of rats chronically treated with the antipsychotic drugs haloperidol or risperidone found that mGluR5 expression in the frontal cortex was not altered, although significant changes were observed in subcortical regions, such as the caudate nucleus [22]. Second, tissue samples were only obtained from white male subjects. Thus, the presence of any potential sex-related or ethnic variation in mGluR5 binding or expression cannot be ruled out. The mutation responsible for FXS is known to affect men and women at equal rates, but owing to lyonization or X chromosome inactivation, FMRP levels are higher in women, with commensurately fewer and less severe physical, cognitive, and behavioral phenotypes [1]. Third, most tissue samples were from full mutation cases of FXS; only four samples were from FXS carriers. When FXS carrier samples were excluded from the analysis, both mGluR5 density and expression were higher, but only mGluR5 density was marginally significant between FXS and control samples. Unlike FXS patients with full mutation, premutation carriers are known to express low levels of functional FMRP. However, the carriers are known to have increased transcription of FMR1 mRNA, causing RNA toxicity by sequestering several proteins to form intracellular inclusion bodies [2]. These inclusion bodies have been found to contain several RNA-binding proteins, such as hnRNPA2/B1, CUGBP1 and Sam68 [23,24]. Currently, it is not known whether RNA toxicity in carriers is associated with sequestration of receptor protein, such as mGluR5. Thus, the current finding of mGluR5 density or expression warrants further study in a greater number of subjects who are only FXS full mutation or FXS carriers. Furthermore, for most samples, neither information on the number of CGG repeats nor the methylation status in the *FMR1* gene was available. The availability of this information would have allowed us to assess the correlation between methylation or CGG repeats and mGluR5 binding or expression, given that epigenetic modification of the *FMR1* gene has been implicated in differential therapeutic response to mGluR5 antagonists in individuals with FXS [20]. Finally, owing to the limited availability of tissue samples, we were only able to evaluate mGluR5 measures in the prefrontal cortex of humans; other subcortical regions known to contribute to the various cognitive and psychomotor effects observed in FXS (for example, hippocampus, amygdala, and cerebellum) might have yielded different mGluR5 measures.

Despite these limitations, the marginally increased mGluR5 receptor density and expression observed in this study in individuals with FXS provide a strong rationale for measuring mGluR5 in living patients using positron emission tomography (PET) [25,26]. Such studies could address many questions raised in the postmortem study. First, PET could determine whether increased mGluR5 binding is present in live subjects and in all brain areas, including the prefrontal cortex. However, PET typically does not separately measure receptor density (*B*_max_) and affinity (1/*K*_D_). Instead, PET uses low tracer doses of the radioligand, which measures the product of these two variables, called the binding potential (BP = *B*_max_/*K*_D_). In our postmortem study, both BP and *B*_max_ were increased (16% and 17%, respectively) with marginal statistical significance (*P* = 0.058 and 0.092, respectively) for FXS patients compared with controls (Table 2). Even after excluding four FXS carriers, both BP and *B*_max_ were increased (17% and 19%, respectively) with marginal statistical significance (*P* = 0.071 and 0.094, respectively) for FXS patients compared with controls. Because PET can only measure BP, based on 80% power and two-tailed *P* ≤ 0.05, the required sample size for PET to detect a significant elevation in BP in the prefrontal cortex of FXS patients would be approximately 45 subjects. Second, the records from the postmortem samples contained inadequate information about prior drug treatment, which may have upregulated mGluR5. By selecting live, unmedicated subjects, PET imaging can more directly assess the possible confound of concomitant medications. Third, baseline mGluR5 levels could be correlated to any response to treatment with mGluR5 negative allosteric modulators. For example, would those subjects with the highest elevation of mGluR5 have the greatest response to this antagonist treatment?

## Conclusions

This study was the first to identify upregulation of mGluR5 density and expression in the prefrontal cortex of FXS patients or carriers compared to an age- and sex-matched control group. This is consistent with several studies in FXS model mice that postulate that the syndromic features of FXS are caused by an upregulated mGluR5 signaling pathway. Although the sample size was relatively small and the results could be secondary to prior medication treatment, these initial findings provide strong rationale for measuring mGluR5 in live patients using PET. Such *in-vivo* studies could measure mGluR5 in all brain regions; the results could also be correlated with treatment response to mGluR5 negative allosteric modulators.

## Abbreviations

BP: Binding potential; FMR1: Fragile X mental retardation 1; FMRP: Fragile X mental retardation protein; FXS: Fragile X syndrome; FXTAS: Fragile X-associated tremor ataxia syndrome; HPLC: High-performance liquid chromatography; KO: Knockout; mGluR: Metabotropic glutamate receptor; mGluR5: Metabotropic glutamate receptor type 5; MPEP: 2-methyl-6-(phenylethynyl)-pyridine; MPEPy: 3-methoxy-5-pyridin-2-ylethynylpyridine; PET: Positron emission tomography.

## Competing interests

MFB declares a financial interest in Seaside Therapeutics. TGL, EKO, MF, KJJ and RBI report no biomedical financial interests. The authors declare that they have no competing interests.

## Authors’ contributions

TGL participated in the study design, performed homogenate binding assays and statistical analysis, and drafted the manuscript. EKO carried out immunoblotting and statistical analysis and drafted the manuscript. KJJ participated in study design and homogenate binding assays. MFB, RBI, and MF conceived the study, participated in its design and coordination and helped to draft the manuscript. All authors read and approved the final manuscript.
